# Cortical regulation of dopaminergic neurons: role of the midbrain superior colliculus

**DOI:** 10.1152/jn.00329.2013

**Published:** 2013-11-13

**Authors:** C. Bertram, L. Dahan, L. W. Boorman, S. Harris, N. Vautrelle, M. Leriche, P. Redgrave, P. G. Overton

**Affiliations:** ^1^Department of Psychology, University of Sheffield, Western Bank, Sheffield, United Kingdom; and; ^2^UPS, Centre de Recherches sur la Cognition Animale, Université de Toulouse, Toulouse, France

**Keywords:** dopamine, basal ganglia, electrophysiology, salience

## Abstract

Dopaminergic (DA) neurons respond to stimuli in a wide range of modalities, although the origin of the afferent sensory signals has only recently begun to emerge. In the case of vision, an important source of short-latency sensory information seems to be the midbrain superior colliculus (SC). However, longer-latency responses have been identified that are less compatible with the primitive perceptual capacities of the colliculus. Rather, they seem more in keeping with the processing capabilities of the cortex. Given that there are robust projections from the cortex to the SC, we examined whether cortical information could reach DA neurons via a relay in the colliculus. The somatosensory barrel cortex was stimulated electrically in the anesthetized rat with either single pulses or pulse trains. Although single pulses produced small phasic activations in the colliculus, they did not elicit responses in the majority of DA neurons. However, after disinhibitory intracollicular injections of the GABA_A_ antagonist bicuculline, collicular responses were substantially enhanced and previously unresponsive DA neurons now exhibited phasic excitations or inhibitions. Pulse trains applied to the cortex led to phasic changes (excitations to inhibitions) in the activity of DA neurons at baseline. These were blocked or attenuated by intracollicular administration of the GABA_A_ agonist muscimol. Taken together, the results indicate that the cortex can communicate with DA neurons via a relay in the SC. As a consequence, DA neuronal activity reflecting the unexpected occurrence of salient events and that signaling more complex stimulus properties may have a common origin.

dopamine-mediated transmission has been implicated in a number of human clinical disorders as well as in a wide range of normal brain functions. Typically, dopaminergic (DA) neurons exhibit a stereotyped short-latency, short-duration population response to unpredicted stimuli in a variety of modalities that are salient by virtue of their novelty, intensity, or reward value (Freeman and Bunney 1987; Horvitz et al. 1997; Schultz 1998). In the case of vision, we have previously shown that visual information is provided to DA neurons by the midbrain superior colliculus (SC) (Comoli et al. 2003; Dommett et al. 2005). The SC is a primitive subcortical visual structure with limited perceptual processing capabilities. Hence, visual neurons in the primate colliculus are sensitive to the onset, offset, and movement of stimuli, but the majority are insensitive to shape, contrast, orientation, velocity, and direction of movement (Goldberg and Wurtz 1972; Schiller and Koerner 1971). Furthermore, the SC does not receive an input from color opponent cells in the retina (Schiller and Malpeli 1977).

However, despite this limited processing capacity in the colliculus, DA neurons in the primate appear able to distinguish between complex visual stimuli and can reflect the magnitude and probability of obtaining the reward with which they are associated (Fiorillo et al. 2003; Morris et al. 2004; Nomoto et al. 2010; Tobler et al. 2005). Closer analysis of these responses, however, suggests that the response of DA neurons to complex visual stimuli is actually biphasic. The truly discriminatory phase of the DA response has a peak latency around 80–100 ms after an earlier, more invariant response feature (Hudgins et al. 2009; Morris et al. 2004; Nomoto et al. 2010). Indeed, more generally, it has been proposed that short-latency responses in DA neurons reflect the sensory intensity of the inducing stimulus while the later phases reflect motivational value (Fiorillo et al. 2013a). In relation to complex visual stimuli, given the rather primitive visual processing capabilities of the SC, the natural assumption would be that the shorter-latency component of the DA response is of collicular origin while the longer-latency discriminatory phase has its origin elsewhere in the brain. Sophisticated visual processing required to discriminate between complex stimuli most likely involves the visual cortex (Orban 2008; Thorpe and Fabre-Thorpe 2001; Zeki 1993); however, cortical inputs to DA neurons are relatively sparse (Frankle et al. 2006; Watabe-Uchida et al. 2012). In contrast, the cortical mantle projects heavily to the SC in a wide range of species (monkey: Fries 1984; cat: Harting et al. 1992; rat: Comoli et al. 2012). The large cortical projection to the SC suggests that the cortical information that may be signaled by the later phase of the sensory response in DA neurons could arise via a relay in the SC. Indeed, cortically dependent responses in the SC are temporally delayed with respect to those arising from more direct sensory activation (Cohen et al. 2008; White et al. 2009). The possibility that the cortex can communicate with DA neurons via the SC was tested in the present study by using electrical stimulation to directly activate the cortex while recording the activity of DA neurons in the presence of pharmacological manipulation of the SC.

For a number of reasons, the whisker barrel cortex was chosen as the locus of stimulation in the present study: *1*) Dopaminergic neurons are exquisitely sensitive to whisker deflection in the awake rat (Freeman et al. 1985). *2*) Inputs from the somatosensory cortex terminate primarily in the intermediate layers of the SC (Wise and Jones 1977), the lateral aspect of which contain a concentration of neurons that project directly to DA neurons in the ventral midbrain (Comoli et al. 2003). *3*) Lateral intermediate layer neurons respond vigorously to whisker deflection in the rat (Hemelt and Keller 2007). Although the barrel cortex constitutes an early stage of cortical sensory processing, it is clear from the research literature that cells there encode complex stimulus characteristics and can do so adaptively (e.g., Diaz-Quesda and Maravall 2008; Jacob et al. 2008). Hence even this primary sensory area is in a position to potentially communicate highly processed stimulus-related information to DA neurons. To confirm that activation induced by electrical stimulation of the cortex was confined within the barrel region, optical imaging was performed to map the extent of the activation.

## MATERIALS AND METHODS

All aspects of these studies were performed with Home Office approval under section 5(4) of the Animals (Scientific Procedures) Act 1986, and experimental protocols received prior approval from the Institutional Ethics Committees.

### Electrophysiology

#### Recording and injection procedure.

Twenty-two male Hooded Lister rats (288–515 g) were anesthetized with an intraperitoneal injection of urethane (ethyl carbonate, 1.25 g/kg as a 25% aqueous solution; additional doses of 25 mg/kg were administered if required) and mounted in a stereotaxic frame with the skull level. Body temperature was maintained at 37°C with a thermostatically controlled heating blanket. Craniotomies were performed to allow unilateral access to the barrel cortex and SC, and to DA neurons in the substantia nigra pars compacta (SNc), and a concentric bipolar electrode (NEX-100, Rhodes Medical Instruments, Woodland Hills, CA) was inserted into the former structure (1.6–3.3 mm posterior to bregma, 4.2–5.4 mm lateral to midline, and 1.5–1.8 mm ventral to the brain surface).

Extracellular single-unit recordings were made from DA neurons with glass microelectrodes with a tip diameter of ∼1.0–2.5 μm (impedances 6–10 MΩ, measured at 135 Hz in 0.9% NaCl). Electrodes were filled with 2 M saline and 2% pontamine sky blue (BDH Chemicals, Poole, UK). After manufacture, the electrode was lowered to a position just dorsal to the SNc with a hydraulic microdrive (model 650, David Kopf Instruments), ipsilateral to the cortical stimulating electrode. An angled (35°) contralateral approach (beginning 2.2–4.4 mm lateral to midline on the opposite side of the brain) was used to avoid collision with a second recording electrode inserted vertically into the SC (see below). All subsequent stages of the experiment were conducted in the dark.

Extracellular multiunit recordings were made from SC neurons ipsilateral to the DA recording and cortical stimulating electrodes with a Parylene C-coated tungsten electrode (A-M Systems, Carlsborg, WA) coupled to a 30-gauge stainless steel injector filled with either the GABA_A_ receptor antagonist bicuculline methiodide (100 ng/μl in 0.9% saline; Sigma-Aldrich, Poole, UK) or the GABA_A_ receptor agonist muscimol (200 ng/μl in saline; Sigma-Aldrich). Lateral separation between the electrode and the tip of the injector was 0.2–0.5 mm, with the electrode positioned 0.5 mm forward of the injector. Electrophysiological responses were determined while the electrode-injector assembly was lowered into the SC in the presence of a whole field light flash (0.5 Hz, 10-ms duration) from a yellow LED (30 lux, 585–590 nm) positioned 5 mm from the contralateral eye, which was sutured open. With the characteristically vigorous visual response of the superficial layers of the SC as a positional cue, the electrode was lowered into the lateral intermediate layers (6.0–6.8 mm caudal to bregma, 1.6–2.6 mm lateral to midline, 4.7–5.7 mm ventral to the brain surface). Intracollicular microinjections were made (0.5 μl at a rate of 0.5 μl/min) via a 10-μl Hamilton syringe mounted on an infusion pump connected to the injector by a length of plastic tubing.

Spike-related potentials were amplified, band-pass filtered (−3 dB points: 200 Hz–4 kHz for single-unit recordings, 400 Hz–16 kHz for multiunit recordings), digitized at 20 kHz, and recorded directly onto computer disk with a 1401 Plus data acquisition system [Cambridge Electronic Design (CED) Systems, Cambridge, UK] running CED data capture software (Spike 2).

Once the SC electrode/injector had been positioned, the DA electrode was lowered until a putative DA neuron was identified on the basis of standard criteria (Grace and Bunney 1983; Ungless et al. 2004): long-duration (>2.0 ms) biphasic or triphasic action potential, with an initial phase exceeding >1.0 ms in length, a low firing rate (<10 Hz), and a firing pattern that consisted of irregular single spikes or bursts. Dopaminergic neurons were encountered at 4.6–6.0 mm posterior to bregma, 1.8–3.2 mm lateral to midline, and 8.0–9.7 mm ventral to the brain surface. Once encountered, the activity of the cell (and multiunit activity in the SC) was recorded during the application of electrical pulses to the cortex. Stimulation consisted of either single square wave pulses (0.1 ms, 1 mA) or trains of square wave pulses (5 pulses at 150 Hz, 0.1 ms each, 0.6–0.8 mA; designed to mimic the activity of tectally projecting intrinsically bursting pyramidal neurons in the cortex; see Kasper et al. 1994; Rumberger et al. 1998; Tsiola et al. 2003). The timing of the electrical stimuli was jittered by 20% around a mean frequency of 0.25 Hz (single pulses) or 0.5 Hz (pulse trains).

Single-pulse electrical stimulation of the cortex frequently did not elicit changes in the activity of DA neurons at baseline. Hence, when single-pulse stimuli were used, after a period of drug-free response determination (150 trials) a pressure injection of bicuculline was made into the SC to disinhibit the structure (*N* = 13 animals), a paradigm that we have previously shown to convert previously unresponsive DA neurons into neurons that exhibit short-latency responses to light flash stimuli (Dommett et al. 2005). Hence, for comparison, in the single-pulse condition each pulse was preceded 2 s earlier by a whole field light flash. Drug-free responses in DA neurons to cortical stimulation could be elicited more reliably by pulse trains. Therefore, when pulse trains were used, after a period of drug-free response determination (150 trials) an injection of muscimol was made into the SC to inhibit the structure (*N* = 9 animals). The demonstration that responses to cortical stimulation in DA neurons could be facilitated by intracollicular bicuculline and blocked/attenuated by intracollicular muscimol would provide convergent evidence that cortically induced activation of DA neurons occurred via a route of transmission that involves the SC. Electrical stimulation was applied to the cortex throughout the postdrug period, until the effects of the drug wore off in the SC (in the case of bicuculline), the effect of the drug had reached an asymptote (in the case of muscimol), or the DA neuron was lost. After a complete trial with bicuculline, which is relatively short acting in the SC (10–15 min; Dommett et al. 2005), further DA neurons were tested in the same way. Between one and three DA neurons were tested in a single subject. The effect of muscimol in the SC is much longer lasting than that of bicuculline (>90–120 min; Edeline et al. 2002), and so only a single DA neuron was tested in each subject in the muscimol experiments.

#### Histological processing.

In each case, the final recording site of the DA electrode was marked with pontamine sky blue by passing a 27.5-μA cathodal current for 15–25 min with a constant current source (Fintronics, Orange, CT). The recording/injection site in the SC was marked with a small electrolytic lesion (10 μA, 150 s, cathodal current) with the same constant current source. Animals were then killed with an overdose of barbiturate and perfused transcardially with 400 ml of warmed saline (40°C), followed by 400 ml of paraformaldehyde in phosphate buffer (PB, pH 7.4). Brains were removed and postfixed overnight in 4% paraformaldehyde at 4°C before being transferred into sucrose for 36 h. Serial coronal (30 μm) sections were cut in a cryostat. One series of sections was mounted on slides and processed with a Nissl stain (cresyl violet), while a second series was collected in 0.1 M PB and processed for tyrosine hydroxylase (TH) and, in cases where bicuculline had been injected, c-fos immunohistochemistry as previously described (Coizet et al. 2006).

#### Data analysis.

The waveform characteristics of recorded DA neurons were determined off-line from averaged records of typically 60–90 digitized spikes/neuron. These averages were used to determine the width of the action potential according to the criteria of both Grace and Bunney (1983) and Ungless et al. (2004). Once the waveform characteristics of the recorded DA neurons had been assessed, analysis of the responses of these neurons and neurons in the SC to the applied stimuli was facilitated by removal of the artifact associated with electrical stimulation from the data records. To achieve this, a waveform average was constructed for the trials to be analyzed, covering the period 1 ms before to 9 ms after the stimulation. This was then subtracted from the raw data trace. Action potentials of DA neurons were then isolated from background noise by using the WaveMark function in Spike 2 (CED), which marked the temporal position of each action potential as an event. In the case of the SC data, waveform averages were again constructed and subtracted from the raw data. The SC trace was then high-pass filtered with an FIR digital filter (Spike 2, CED; transition gap 1.2 kHz, −3 dB point: 1.069 kHz) and rectified. A spike threshold was set at two standard deviations (SDs) above the mean of the rectified voltage waveform. For pre- vs. postdrug comparisons, the threshold value determined before the drug was held over and applied after the drug to allow drug-induced changes in firing rate to be assessed.

After this preprocessing, peristimulus time interval histograms (PSTHs) were constructed based on DA single-unit and SC multiunit data, with a bin width of 20 ms and 1 ms, respectively; these bin widths, tailored to the cell type, were used to extract quantitative aspects of cell behavior that most closely matched estimates based on visual inspection for the analysis period. PSTHs for DA single-unit data were smoothed with a three-point sliding average. Although smoothing combined with a 20-ms bin width attenuated the variability associated with the low firing rate in DA neurons, some degree of temporal precision in the measurement of responses in the cells had to be sacrificed to achieve this. PSTHs based on DA single-unit and SC multiunit data were then analyzed to determine the following response characteristics before and after an injection of bicuculline/muscimol: *1*) prestimulation baseline activity: the number of spikes per second occurring during the 500 ms prior to the light flash and/or cortical stimulation, divided by the number of trials; *2*) poststimulation baseline activity: the mean number of spikes per second occurring during the 200 ms following the light flash stimulation, divided by the number of trials; *3*) response latency: defined as the time point at which deviations in activity after light flash or electrical stimulation exceeded 1.96 SDs of the baseline activity, sustained for three or more bins in the case of DA neurons (DA neurons were considered to be “responsive” if at least 3 consecutive bins within a period spanning 20–260 ms following the stimulation exceeded the 1.96 SD threshold); *4*) response duration: response offset was defined as the time point at which activity returned to within the threshold values after light flash or electrical stimulation, sustained for two or more bins in the case of DA neurons—response duration was the difference in time between response latency (onset) and offset; *5*) response magnitude: the total number of spikes between response onset and offset, minus the prestimulation baseline activity for excitatory response components and subtracted from the baseline mean for inhibitory response components, divided by the number of trials.

When considering the effect of collicular bicuculline on the responses of DA neurons to electrical stimulation, given that the action of bicuculline in the SC is short lived (Dommett et al. 2005), DA and collicular neuronal activity was analyzed for the period over which the drug affected SC activity. This period was defined as starting when the poststimulation baseline activity exceeded a threshold of +2 SDs of prebicuculline levels for two consecutive sets of 10 stimulations after collicular bicuculline and was defined as ending when two consecutive sets of 10 stimulations fell below the same threshold. Since the effects of muscimol in the SC are much longer lasting (see, e.g., Edeline et al. 2002), the effects of this drug were determined for the whole postdrug period. Analysis focused on the later phases of the postdrug period because of the slow diffusion rate of the drug (Edeline et al. 2002). Quantitative differences in pre- vs. postdrug response parameters were assessed with Student's or Welch's *t*-tests. The latter was used if samples had unequal variance or sizes, and the Wilcoxon signed-rank test was used where data were nonnormally distributed. Statistical tests were confined to situations in which group sizes exceeded four per condition. In all cases, the accepted significance level was *P* < 0.05, two-tailed.

### Two-Dimensional Optical Imaging Spectroscopy

To assess the spread of cortical activation induced by the electrical stimulation protocols used in the present study, two-dimensional optical imaging spectroscopy (2D-OIS) was used to obtain a two-dimensional activation map evoked by the stimulation, based on changes in local tissue hemodynamics. The hemodynamic response evoked by direct electrical stimulation of the barrel cortex was compared to the hemodynamic response elicited by whisker pad stimulation.

Three additional female Hooded Lister rats (230–330 g) were anesthetized with an intraperitoneal injection of urethane (1.25 g/kg), and additional doses of 25 mg/kg were administered if required. Our methodology for 2D-OIS has been described in detail elsewhere (Boorman et al. 2011). Briefly, animals were tracheotomized and artificially ventilated, and the left and right femoral arteries and veins were cannulated for measurement of mean arterial blood pressure and for the infusion of phenylephrine. Physiological parameters were maintained within normal ranges. Platinum stimulating electrodes insulated to within 2 mm of the tip were inserted in a posterior direction between rows A/B and C/D of the left whisker pad of the rat. The animals were placed in a stereotaxic frame with the skull level, and the skull overlying the somatosensory cortex was thinned to translucency. A circular plastic “well” (20-mm diameter) was positioned over the thinned area, attached with dental cement, and filled with saline. A small hole was drilled in the thinned skull above the barrel cortex, and a bipolar stimulating electrode (NEX-100, Rhodes Instruments) was introduced perpendicularly to a depth of 1.5 mm.

For 2D-OIS, a Dalsa 1M30P camera recorded the images (with each pixel representing 75 × 75 μm) and a Lambda DG-4 high-speed filter changer (Sutter Instrument, Novato, CA) was used to sequentially expose the thinned cranial window to four different wavelengths of visible light. Spectral analysis was used to produce two-dimensional images over time of oxyhemoglobin (HbO_2_), deoxyhemoglobin (Hbr), and total blood volume (Hbt). The effects of 60 trials of direct electrical stimulation of the cortex were assessed with both single pulses and trains of pulses (each single pulse or train was separated from the next by 26 s), with the same stimulation parameters as used in the electrophysiology experiments (single pulses: 0.1 ms, 1 mA; trains of pulses: 5 pulses at 150 Hz, 0.1 ms each, 0.6 mA). The hemodynamic changes evoked by electrical stimulation of the barrel cortex were compared to the effects of whisker pad stimulation at 5 Hz (1.2 mA for 16 s). A stimulation frequency of 5 Hz is known to result in the greatest magnitude of hemodynamic responses in the somatosensory cortex of the anesthetized rat (Martin et al. 2006).

#### Data analysis.

The first stage of the analysis was to determine the center of an area of activation with the general linear model (GLM) statistical parametric mapping (SPM) approach (Friston et al. 1991). The time series of each pixel was compared to a design matrix consisting of a DC offset and a square wave representing the hemodynamic response function. This allowed voxel-by-voxel calculation of activation *z* scores. The spatial distribution of activation was determined by plotting the region of activation exceeding a *z*-score threshold. The center point of this region was then used for the second stage in the analysis—determining the spatial spread of the hemodynamic response. The area around the center of activation was divided into a series of concentric circular regions (approximate inter-ring distance = 0.25 mm). The change in total hemodynamic concentration for the pixels inside each concentric ring was integrated, providing a measure of the hemodynamic response at a given distance away from the center of activation. The hemodynamic response in each ring was then averaged across animals, and the average response for each ring was plotted for electrical whisker pad stimulation and cortical stimulation to produce distance-decay curves.

## RESULTS

### Transcortical Spread of Activation Induced by Cortical Stimulation

**Fig. 1.**
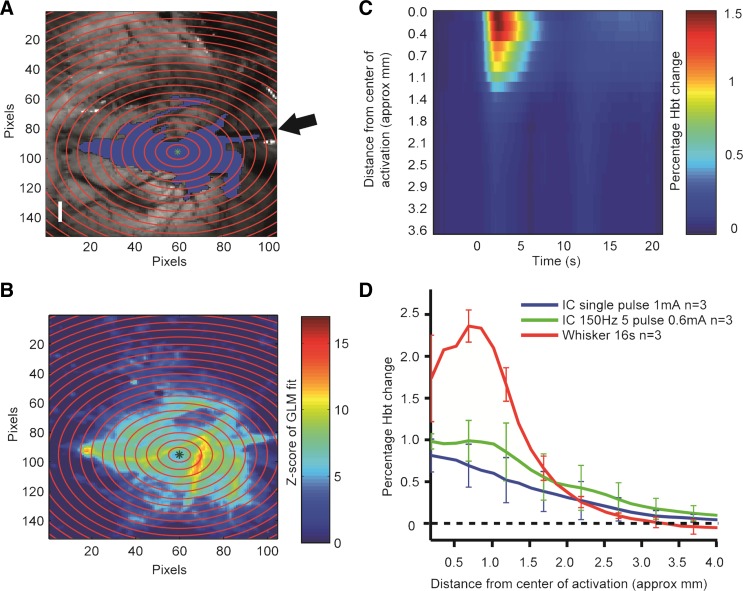
Cortical hemodynamic responses to direct electrical stimulation and electrical stimulation of the whisker pad. *A*: in vivo camera image of the cortical surface vasculature viewed through a thinned cranial window. A small hole drilled through the thinned skull allowed a stimulating electrode (arrow) to be inserted into the barrel cortex. Single-pulse stimulation (0.1 ms, 1 mA) produced a localized increase in blood volume (Hbt), as indicated by the blue shaded area [general linear model (GLM) model fit, *z*-score threshold at 50%]. Scale bar, 1 mm. *B*: concentric rings centered on the geometrical midpoint of the area of activation were used to analyze the spread of activation (approximate inter-ring distance = 0.25 mm). The magnitude of the evoked hemodynamic response within each ring at the temporal peak of activation forms the basis for *B–D*. Key represents the GLM model fit as a *z* score. *C*: temporal and spatial spread of activation after single-pulse stimulation of the barrel cortex. Key represents % change in Hbt relative to baseline. Stimulation occurred at *time 0*. *D*: spatial spread of activation (mean change in Hbt) in the cortex induced by single-pulse stimulation and pulse train stimulation (5 pulses at 150 Hz, 0.1 ms each, 0.6 mA) of the barrel cortex and electrical stimulation of the whisker pad (5 Hz, 1.2 mA for 16 s).

**Fig. 2.**
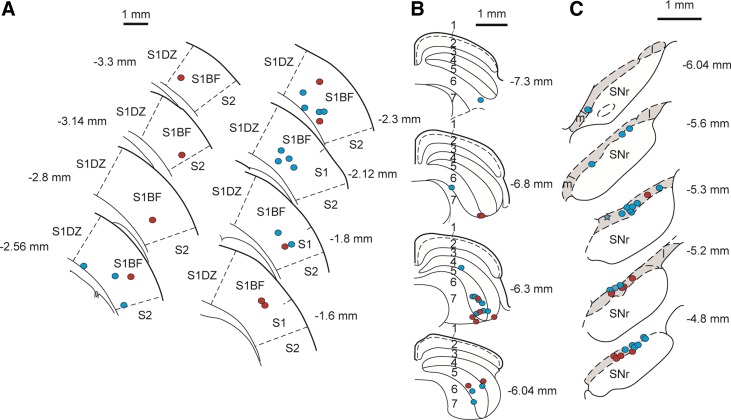
Reconstructed plots of stimulation, recording, and injection sites. *A*: plots of stimulation sites in the cerebral cortex. Colored circles indicate the position of the electrode tips in animals in which pulse trains were applied to the cortex (red) or single pulses were applied to the cortex (blue). S1, primary somatosensory cortex; S1BF, primary somatosensory cortex, barrel field; S1DZ, primary somatosensory cortex, dysgranular region; S2, secondary somatosensory cortex. *B*: reconstructed plots of recording/injection sites in the superior colliculus. Colored circles indicate the position of the recording/injection sites in animals in which muscimol was injected into the colliculus (red) or bicuculline was injected into the colliculus (blue). The layers are labeled as follows: 1, zonal layer; 2, superficial gray layer; 3, optic layer; 4, intermediate gray layer; 5, intermediate white layer; 6, deep gray layer; 7, deep white layer. *C*: reconstructed plots of recording sites in the substantia nigra pars compacta (SNc). Colored circles indicate the position of the recording sites in animals in which pulse trains were applied to the cortex (red) or single pulses were applied to the cortex (blue). The point marked with a star represents the location of 2 recorded dopaminergic neurons. The SNc is indicated by shading. m, Substantia nigra medial part; l, substantia nigra lateral part; SNr, substantia nigra pars reticulata. In *A–C*, electrode positions are reconstructed onto coronal sections, the position of which is given relative to bregma.

2D-OIS was used to assess the extent of spread of activation induced by the electrical stimulation parameters used to stimulate the barrel cortex in the present study. Single-pulse electrical stimulation of the cortex produced a localized region of enhanced activity (Fig. 1, *A* and *B*), which peaked 2.2 ± 0.3 s after stimulus onset and decreased rapidly both temporally and spatially (Fig. 1, *C* and *D*), having a radius at its peak of ∼2.0 mm (Fig. 1*D*). The response produced by single-pulse stimulation had a smaller peak amplitude, but a similar extent of spread, than whisker pad stimulation (Fig. 1*D*). The hemodynamic response elicited by a train of pulses had an extent of spread similar to that elicited by single pulses but had a greater peak amplitude (Fig. 1*D*). All stimulating electrode tips in the present study were located in the gray matter of the barrel cortex (Fig. 2*A*), which occupies ∼4.3 mm × 4.0 mm medio-laterally and rostro-caudally in these dimensions (Paxinos and Watson 2004); hence activation is likely to have been largely confined to the barrel cortex in all animals. Significant inclusion of the underlying white matter is contraindicated by the absence of activation of adjacent areas of the cortex within the thinned window (Fig. 1*A*).

### Cortically Evoked Activation of SC

**Fig. 3.**
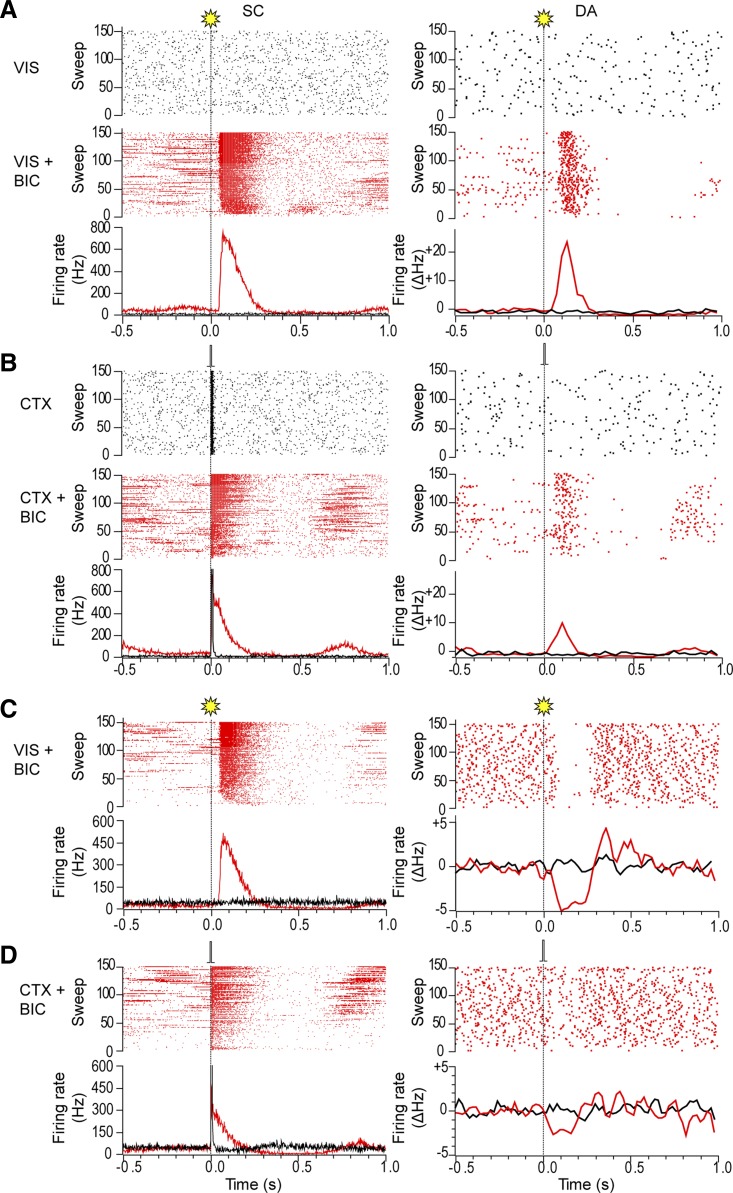
Response of the superior colliculus and dopaminergic neurons to light flash stimuli and electrical stimulation of the barrel cortex. *A*: raster displays (*top*) and peristimulus time interval histograms (PSTHs; *bottom*) show that deep layer collicular neurons (SC) and a simultaneously recorded dopaminergic (DA) neuron in this animal were initially unresponsive to regular (0.5 Hz) whole field light flash stimuli (VIS; vertical dotted line). After a collicular microinjection of bicuculline (VIS+BIC), both collicular neurons and the DA neuron were excited at short latency by visual stimulation. The PSTH for the DA neuron shows the 3-point smoothed change in firing rate from baseline (ΔHz). *B*: raster displays and PSTHs show that collicular neurons exhibited a short-latency excitatory response to single-pulse electrical stimulation of the barrel cortex (CTX; 0.1 ms, 1 mA, 0.5 Hz; vertical dotted line) whereas (in common with the majority of DA neurons) a simultaneously recorded DA neuron in this animal was unresponsive. After a collicular microinjection of bicuculline (CTX+BIC), the collicular response to cortical stimulation was enhanced and the DA neuron was now excited at short latency by the stimulation. As well as short-latency excitations, light flash stimuli (*C*) and single-pulse electrical stimulation of the barrel cortex (*D*) could also induce short-latency inhibitions in DA neurons after bicuculline.

**Fig. 4.**
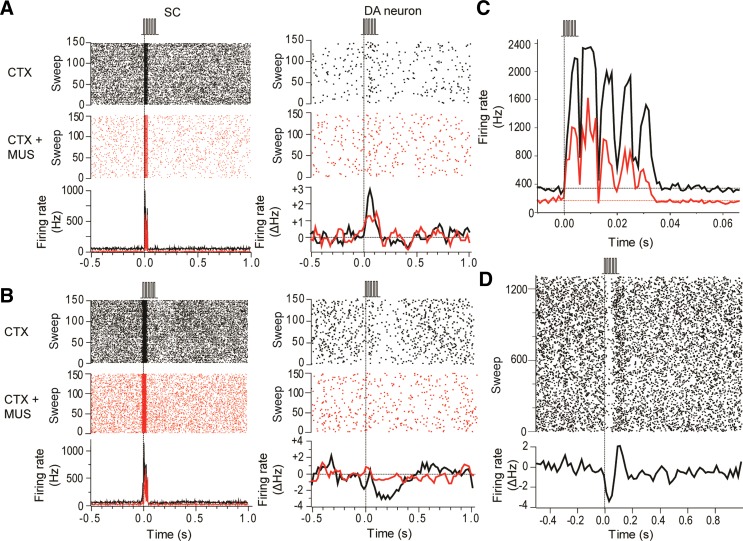
Intracollicular muscimol administration suppressed collicular and dopaminergic responses to cortical stimulation. *A*: raster displays (*top*) and PSTHs (*bottom*) show that collicular neurons (SC) in this animal exhibited a short-latency excitatory response to pulse trains applied to the barrel cortex (CTX; 0.1 ms, 0.6 mA; vertical dotted line). Likewise, a simultaneously recorded DA neuron showed a short-latency excitatory response to the pulse trains. After a collicular microinjection of muscimol (CTX+MUS) the collicular response to cortical stimulation was attenuated, as was the response of the DA neuron. The PSTH for the DA neuron shows the 3-point smoothed change in firing rate from baseline (ΔHz). *B*: as well as excitations, pulse trains applied to the barrel cortex could induce short-latency inhibitions in DA neurons. In the example shown here, intracollicular muscimol eliminated the DA neuron's response to cortical stimulation. *C*: trains of electrical stimuli applied to the barrel cortex produced excitatory responses in the SC to each pulse in the train (black trace). Intracollicular administration of muscimol reduced baseline activity and depressed the responses to stimulation (red trace). *D*: raster display (*top*) and PSTH (*bottom*) of a representative case showing that electrical stimulation of the barrel cortex with pulse trains (5 pulses at 150 Hz, 0.1 ms each, 0.6 mA) produced temporally stable responses in DA neurons.

As expected from our previous work (Coizet et al. 2009; Dommett et al. 2005), neurons in the SC deep layers (for brevity, we refer to all layers below the superficial layers as “deep” here and below) were unresponsive to whole field light flash stimuli in urethane-anesthetized animals (Fig. 3*A*). However, single-pulse electrical stimulation of the barrel cortex produced very short-latency, short-duration excitatory responses in the deep layers of the colliculus, with low temporal variability (Fig. 3*B*; Table 1). Pulse trains also produced very short-latency, temporally stable excitatory responses in the colliculus (Fig. 4, *A–C*; see Table 3), which had significantly longer durations than those following single-pulse stimulation (*t*[13.8] = 3.4, *P* < 0.05) but similar onset latencies (W = 131.5, *P* > 0.05; measured with respect to 1st stimulus in the train). The excitatory response to pulse trains consisted of discrete, progressively decrementing responses to each pulse in the train, and response size lessened across the train, such that the responses to earlier stimuli in the train were larger than those to later stimuli in the train (Fig. 4*C*; *t*[8] = 2.81, *P* < 0.05; assessed by measuring the activity in the 6.5 ms following the 2nd and last pulse in the train).

**Table 1.** Effects of intracollicular bicuculline on collicular and dopaminergic responses to cortical stimulation using single pulses

	SC Multiunit		DA(1) Single Unit		DA(2) Single Unit	
	Prebicuculline	Postbicuculline	Prebicuculline	Postbicuculline	Prebicuculline	Postbicuculline
Baseline, Hz	226.9 ± 10.2	318.2 ± 33.5†	3.3 ± 0.4	2.8 ± 0.4	2.8 ± 0.4	2.9 ± 0.5
Latency, ms	2.4 ± 0.3	2.6 ± 0.4		30 ± 9.4	56.9 ± 14.8	17.5 ± 5.1*
Duration, ms	18.2 ± 1.6	75.0 ± 12.3‡		138 ± 23.1	78.8 ± 11.7	125.8 ± 21.2
Magnitude	8.8 ± 1.3	28.3 ± 5.0‡		0.6 ± 0.2	0.1 ± 0.0	0.4 ± 0.2

Values are means ± SE for parameters (baseline firing rate, response latency, response duration, and response magnitude, defined as the mean number of spikes per stimulus between response onset and response offset) of multiunit responses measured in the superior colliculus (SC) and single-unit responses measured in dopaminergic (DA) neurons to electrical stimulation of the barrel cortex using single pulses. In the case of DA neuron responses, cells have been subdivided into 2 groups: cells that were unresponsive [DA(1)] and cells that were responsive [DA(2)] to cortical stimulation in the absence of bicuculline.

* *P* < 0.05,

† *P* < 0.01,

‡ *P* < 0.001 with respect to predrug.

**Fig. 5.**
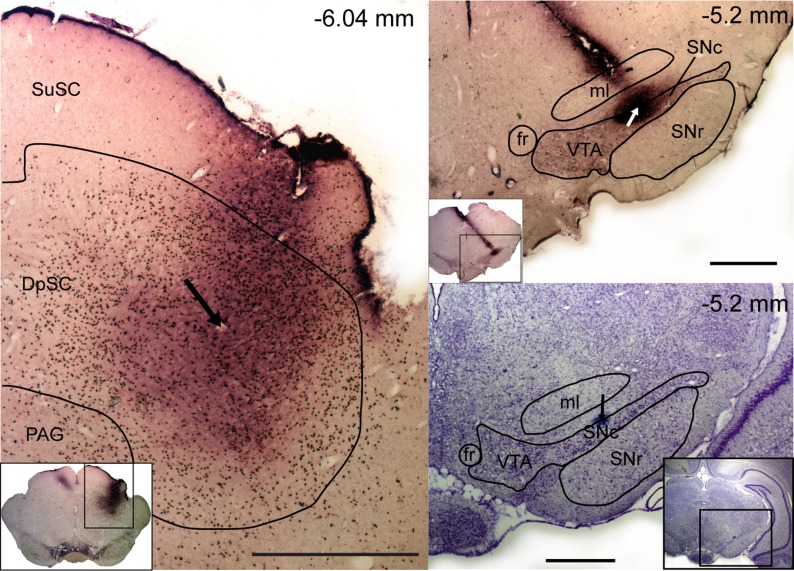
Location of the recording and injection sites in representative animals. *Left*: a coronal section at high and low (*inset*) magnification, showing the superior colliculus processed with tyrosine hydroxylase (TH) and c-fos immunohistochemistry. The section shows fos-like immunolabeling (black dots) in the colliculus as a result of neural activity induced by an injection of bicuculline. An electrolytic lesion at the recording/injection site is indicated by an arrow. *Right*: coronal sections at high and low (*insets*) magnification, processed with TH immunohistochemistry (*top*) and cresyl violet (*bottom*). *Top*: TH immunolabeling (purple cells and processes) in the ventral midbrain and the electrode track and recording site (arrow) in the SNc. *Bottom*: the site can be seen again as a blue dot (indicated by an arrow). The position of all sections is given relative to bregma. Scale bars, 1 mm. SuSC, superficial layers of the SC (zonal layer, superficial gray layer, and optic layer); DpSC, deep layers of the SC (intermediate gray and intermediate white layers, deep gray/white layers); PAG, periaqueductal grey; VTA, ventral tegmental area; ml, media lemniscus; fr, fasciculus retroflexus.

Local injections of the GABA_A_ receptor antagonist bicuculline into the deep layers of the SC (see Fig. 5 for a typical recording/injection site in the colliculus and Fig. 2*B* for reconstructed plots of all sites) produced widespread disinhibition within the colliculus, as evidenced by an increase in baseline firing rate (Tables 1 and 2) and extensive expression of c-fos product, a marker for neuronal activation (Herrera and Robertson 1996; see Fig. 5). After intracollicular bicuculline, collicular neurons exhibited a short-latency, phasic excitation in response to the light flash (Fig. 3, *A* and *C*; Table 2; cf. Coizet et al. 2009; Dommett et al. 2005). At the same time, there was a significant increase in the magnitude and duration of the phasic response to single-pulse stimulation of the cortex, although the onset latency of the response did not change (Fig. 3, *B* and *D*; Table 1). The onset latency of the response to light flash stimuli was significantly longer than that to cortical stimulation (Tables 1 and 2; W = 576, *P* < 0.05).

**Table 2.** Effects of intracollicular bicuculline on collicular and dopaminergic responses to light flash stimuli

	SC Multiunit		DA Single Unit	
	Prebicuculline	Postbicuculline	Prebicuculline	Postbicuculline
Baseline, Hz	220.0 ± 9.9	318.4 ± 33.1†	3.2 ± 0.4	3.5 ± 0.5
Latency, ms		46.0 ± 2.3		86.5 ± 5.3
Duration, ms		172.4 ± 14.6		164.7 ± 14.6
Magnitude		77.8 ± 10.9		1.0 ± 0.2

Values are means ± SE for parameters (baseline firing rate, response latency, response duration, and response magnitude, defined as the mean number of spikes per stimulus between response onset and response offset) of multiunit responses measured in the SC and single-unit responses measured in DA neurons in response to light flash stimuli, before and after intracollicular injections of bicuculline.

† *P* < 0.01 with respect to predrug.

In contrast to the effects of bicuculline on collicular responses to cortical stimulation, intracollicular injection of the GABA_A_ agonist muscimol produced a significant reduction in the magnitude of the responses to stimulus trains (Fig. 4*C*; Table 3). Again, onset latency did not significantly change with the application of muscimol, whereas there was a significant reduction in baseline firing rate (Table 3).

**Table 3.** Effects of intracollicular muscimol on collicular and dopaminergic responses to cortical stimulation using pulse trains

	SC Multiunit		DA(1) Single Unit		DA(2) Single Unit	
	Premuscimol	Postmuscimol	Premuscimol	Postmuscimol	Premuscimol	Postmuscimol
Baseline, Hz	286.6 ± 50.2	95.9 ± 33.1†	2.3 ± 1.0	1.5 ± 0.7	3.7 ± 1.1	4.1 ± 0.9
Latency, ms	1.6 ± 0.2	1.7 ± 0.4	87.8 ± 53.2		87.8 ± 75.3	108.8 ± 35.5
Duration, ms	34.9 ± 2.5	28.6 ± 3.6	153.3 ± 60.3		153.3 ± 85.2	153.8 ± 44.8
Magnitude	16.9 ± 6.3	10.0 ± 5.1*	0.4 ± 0.2		0.3 ± 0.2	0.2 ± 0.1

Values are means ± SE for parameters (baseline firing rate, response latency, response duration and response magnitude, defined as the mean number of spikes per stimulus between response onset and response offset) of multiunit responses measured in the SC and single-unit responses measured in DA neurons to electrical stimulation of the barrel cortex using pulse trains. In the case of DA neuron responses, cells have been subdivided into 2 groups: cells that were unresponsive [DA(1)] and cells that were still responsive [DA(2)] to cortical stimulation in the presence of muscimol.

* *P* < 0.05,

† *P* < 0.01 with respect to predrug.

### Cortically Evoked Activation of DA Neurons

All putative DA neurons sampled in the present study had firing rates (3.1 ± 0.3 Hz) and action potential waveform durations (total duration = 4.2 ± 0.1 ms) that met the criteria of Grace and Bunney (1983) and also met the waveform duration criterion of Ungless et al. (2004; initial duration = 1.4 ± 0.03 ms). These waveform characteristics have been shown to be reliable markers for DA neurons in the SNc (Brown et al. 2009; Ungless and Grace 2012). Furthermore, in all cases the neurons were located in the TH-immunoreactive region of the ventral midbrain corresponding to the SNc (Lindvall and Björklund 1974; see Fig. 2*C*; Fig. 5). All SNc DA neurons tested (*n* = 24) were unresponsive to light flash stimuli in the absence of collicular disinhibition by bicuculline, and the majority (16/24) were also unresponsive to single-pulse electrical stimulation of the cortex. The remaining cells—which did not differ from the unresponsive cells in terms of their position in the SNc or stimulation site in the barrel cortex—showed short-latency phasic responses that had either an initial excitatory component (4/8) or an initial inhibitory component (4/8). The onset latencies of these responses were significantly longer than the corresponding onset latencies in the colliculus (Table 1; *t*[7] = 3.71, *P* < 0.05).

**Fig. 6.**
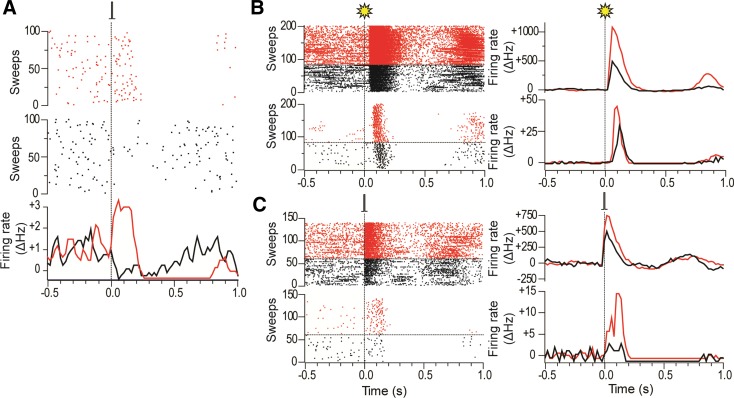
Responses of DA neurons to electrical stimulation of the barrel cortex are labile and interact with responses to visual stimulation. *A*: raster plots (*top* and *middle*) and a PSTH (*bottom*) of a DA neuron that showed a short-latency inhibitory response to single-pulse cortical stimulation of the barrel cortex (0.1 ms, 1.0 mA, 0.5 Hz) in the absence of intracollicular bicuculline (*middle*), which then switched to a short-latency excitatory response in the presence of bicuculline (*top*). The PSTH for the DA neuron shows the 3-point smoothed change in firing rate from baseline (ΔHz). *B*, *top*: raster plot and PSTH of deep layer collicular responses to whole field light flashes in the presence of intracollicular bicuculline. On trials in black flashes were preceded 2 s earlier by electrical stimulation of the barrel cortex, while on trials in red only visual stimulation was used. *Bottom*: raster plot and PSTH of responses in a simultaneously recorded DA neuron. In the presence of cortical stimulation, the response of the DA neuron to visual stimulation in this animal was weaker than when visual stimulation was delivered alone. *C*, *top*: raster plot and PSTH of deep-layer collicular responses to single-pulse electrical stimulation of the barrel cortex in the presence of intracollicular bicuculline. On trials in black electrical stimulation of the barrel cortex was preceded 2 s earlier by whole field light flashes, while on trials in red only electrical stimulation was used. *Bottom*: raster plot and PSTH of responses in a simultaneously recorded DA neuron. In the presence of visual stimulation, the response of the DA neuron to cortical stimulation in this animal was weaker than when stimulation was delivered alone.

**Fig. 7.**
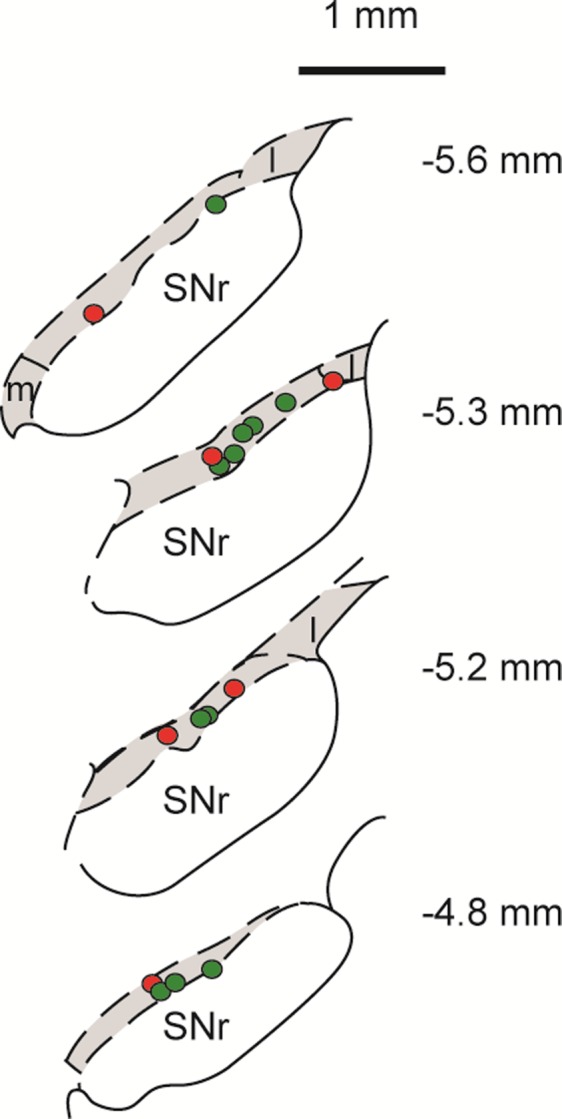
Reconstructed plots of recording sites of dopaminergic neurons showing excitatory or inhibitory initial response components. Colored circles indicate the location of dopaminergic neurons in the SNc showing excitatory (green) or inhibitory (red) initial components in response to cortical stimulation (either single pulses or pulse trains) at baseline. Recording positions are reconstructed onto coronal sections, the position of which is given relative to bregma. The SNc is indicated by shading.

After intracollicular bicuculline, the majority (22/24) of DA neurons exhibited a short-latency, phasic excitatory (14/22) or inhibitory (8/22) response to light flash stimuli (Fig. 3, *A* and *C*; cf. Dommett et al. 2005). Although there was no overall change in the baseline firing rate of DA neurons (Table 1), after bicuculline, 9/16 of the cells that were previously unresponsive to single-pulse cortical stimulation now showed a short-latency excitation (5/16) or inhibition (4/16; Fig. 3, *B* and *D*) following collicular disinhibition. Neurons that already responded in the absence of bicuculline reacted to the treatment by decreasing their response latency (*n* = 3/8; pre 50.0 ± 26.5 ms vs. post 21.7 ± 10.1 ms), becoming unresponsive (*n* = 2/8) or changing the sign of their responses (*n* = 3/8; see Fig. 6*A*). As a consequence of the ability to switch between response types, cells responding to cortical stimulation with either an initial excitation or an initial inhibition do not appear to constitute two separate populations of neurons (unlike DA neurons, which respond to noxious/aversive stimuli with excitations and inhibitions; Brischoux et al. 2009; Matsumoto and Hikosaka 2009; but see Fiorillo et al. 2013b). This conclusion is supported by the fact that those DA neurons that exhibited initial excitations to either single-pulse electrical stimulation (predrug) or pulse trains did not differ from those exhibiting initial inhibitions in terms of baseline activity (excited: 2.8 ± 0.6; inhibited: 3.1 ± 0.6; *t*[14.5] = 0.33, *P* > 0.05) or action potential shape (time from onset of the spike to the 1st trough, excited: 1.3 ± 0.1, inhibited: 1.4 ± 0.1; *t*[14.2] = 1.14, *P* > 0.05). Likewise, there was no evidence of a systematic bias in the location of excited and inhibited cells in the SNc (Fig. 7).

Interestingly, after intracollicular bicuculline, all responsive DA neurons exhibited the same sign response (initial excitation or initial inhibition) to both light flash stimuli and cortical stimulation. As with the colliculus, the onset latency of responses to cortical stimulation was significantly shorter than the onset latency to light flash stimuli [30.0 ms ± 9.4 (combined baseline responsive and newly responsive DA neurons) vs. 86.5 ± 5.3 ms, respectively, *t*[13.4] = 5.21, *P* < 0.05]. In addition, when stimulation in one modality (light or electrical stimulation) temporarily ceased, there was an enhancement of the collicular response to the remaining stimulus (Fig. 6, *B* and *C*). This enhanced response at the collicular level was then reflected in the activity of DA neurons recorded at the same time, which also exhibited an enhanced response (Fig. 6, *B* and *C*).

In contrast to the single-pulse condition, all DA neurons tested (*n* = 9) were responsive to pulse trains applied to the cortex, with cells exhibiting short-latency phasic excitations (6/9) or inhibitions (3/9; Fig. 4, *A* and *B*). Again, the onset latencies of these responses were significantly longer than the corresponding onset latencies in the colliculus (Table 3; *t*[8] = 2.78, *P* < 0.05; measured with respect to the response to the 1st stimulus in the train). Intracollicular muscimol had a profound effect on cortically induced responses in DA neurons. In the absence of muscimol, responses in DA neurons to trains of cortical stimuli were stable over time (Fig. 4*D*). However, after intracollicular muscimol administration, five DA neurons ceased to show a detectable response to cortical stimulation (Fig. 4*B*). In the four cells that remained responsive, muscimol reduced the magnitude of the response (Fig. 4*A*; Table 3). Intracollicular muscimol did not affect the baseline firing rate of DA neurons (*t*[5.2] = 0.74, *P* > 0.05; Table 3).

## DISCUSSION

### Cortical Stimulation and Collicular Responses

2D-OIS showed that with stimulating electrodes in the barrel cortex, the parameters used for stimulation in the present study were likely to have activated an area of cortex that was largely confined to the barrel field. Furthermore, electrical activation of the cortex elicited responses that on a macroscopic scale resembled those elicited by whisker stimulation itself, and hence, although not naturalistic, such activation produces cortical driving the parameters of which fall within those of the system's normal behavior. Furthermore, the lack of evidence for activation of areas of the cortex outside the barrel field suggests a lack of spread of current ventrally to the underlying striatum, which could have generated a change in collicular activity and DA activity via a striato-nigro-collicular route and in DA activity via a direct striato-nigral route.

Although, as in previous work (Coizet et al. 2009; Comoli et al. 2003; Dommett et al. 2005), the deep layers of the SC were unresponsive to whole field light flash stimuli in the absence of bicuculline, these same layers responded with a short-latency, short-duration phasic response to single-pulse electrical stimulation of the barrel cortex. Single-pulse electrical stimulation produced a single, short-latency, temporally stable response in the deep layers of the SC, whereas pulse trains produced a more exaggerated response consisting of a short-latency response following each pulse in the train. The response to single pulses was substantially enhanced after the intracollicular injection of bicuculline and the responses to pulse trains substantially suppressed after intracollicular muscimol. The latency of these responses to cortical stimulation was significantly shorter than the visual responses after bicuculline. Short latency and low temporal variance are commonly used criteria for discriminating monosynaptic from polysynaptic events (see, e.g., Gil et al. 1999; Yoshimura and Jessell 1989), suggesting that the cortically evoked responses are almost certainly monosynaptic in nature. Indeed, the response latencies are very similar to the conduction times in the corticotectal pathway from the primary somatosensory cortex in the rat (median 1.7 ms; Kassel 1982). However, although monosynaptic in nature, there is still the unlikely confounding possibility that they arise as a result of the antidromic activation of cells that provide an orthodromic input to the SC, rather than via the direct activation of cortico-tectal afferents.

Fortunately, only a limited range of structures provide inputs to both the barrel cortex and the SC. The locus coeruleus, dorsal raphe nucleus, and zona incerta all project to the SC (Beitz et al. 1986; Cadusseau and Roger 1985; Dauvergne et al. 2008) and the barrel cortex (Kirifides et al. 2001; Lee et al. 2009; Lin et al. 1990), providing significant noradrenergic, serotonergic, and GABAergic input, respectively. However, the locus coeruleus and dorsal raphe both primarily target the superficial layers (Parent et al. 1981; Wichmann and Starke 1988). Furthermore, the effect of norepinephrine, serotonin, and GABA in the SC is primarily inhibitory (Huang et al. 1993; Mooney et al. 1996; Nicolelis et al. 1992; Sato and Kayama 1983; Tan et al. 1999). Hence the locus coeruleus, dorsal raphe, and zona incerta are not likely to play a role in the generation of the short-latency excitations in the SC we report following barrel cortex stimulation. Instead, the responses most likely reflect the direct orthodromic activation of inputs to the SC deep layers from the cortex.

### Dopaminergic Responses to Cortical Stimulation

All of the presumed SNc DA neurons in the present study met the electrophysiological identification criteria of Grace and Bunney (1983) and the more stringent criterion suggested by Ungless et al. (2004). These waveform characteristics have been shown to be reliable markers for DA neurons in the SNc (Brown et al. 2009; Ungless and Grace 2012). Likewise, all cells were located in the TH-immunoreactive region of the ventral midbrain, corresponding to the A9 DA cell group (Lindvall and Björklund 1974). Non-DA neurons in this region of the brain account for only a small proportion (∼20%; Matsuda et al. 1987) of the total. Nonetheless, in the absence of direct neurochemical identification, the DA nature of our neurons cannot be known with certainty. That said, for the reasons given above, it is probably safe to assume that the overwhelming majority of our neurons were indeed DA.

All SNc DA neurons were unresponsive to light flash stimuli in the absence of local collicular disinhibition with bicuculline, and the majority were unresponsive to single-pulse electrical stimulation of the cortex. In contrast, all cells were responsive to pulse trains applied to the cortex, with cells exhibiting short-latency phasic excitatory or inhibitory responses (as did those cells that were responsive to single-pulse stimulation in the absence of bicuculline). In those cells that responded to cortical stimulation (single pulse or train) at baseline, the latency of responses in DA neurons was significantly longer than the response in the SC, consistent with the transmission of information from the SC to DA neurons in the SNc (see also de Lafuente and Romo 2012). As in our previous work (Dommett et al. 2005), after collicular bicuculline the majority of DA neurons showed a short-latency, phasic response (with an initial excitation or inhibition) to light flash stimuli. Although there was no overall change in the firing rate of DA neurons, after bicuculline 9/16 of the previously unresponsive cells now showed a short-latency phasic response to the single-pulse cortical stimulation. In those cells that exhibited phasic responses to trains of cortical stimuli, intracollicular muscimol abolished the response in the majority of cells and in the remainder decreased the magnitude of the responses.

Neurons showed consistent responses to light flash stimuli and cortical stimulation, responding with either an initial excitation or an initial inhibition to both. Insofar as these responses are driven by the SC, this suggests that the same collicular neurons may be conveying activity related to both stimulation types. This conclusion is further supported by the fact that responses to the two stimulus types tended to interact at both the collicular and nigral levels. It is known that collicular neurons can receive afferent inputs from both cortical and subcortical sources (spinal cord and somatosensory cortex: Rhoades 1980; retina and visual cortex: McIlwain and Fields 1970) and that noncontiguous activation of these afferents results in an inhibitory interaction between them (McIlwain and Fields 1970; Rhoades 1980). In a similar manner in the present study, collicular responses to light flash stimuli were suppressed when preceded 2 s earlier by electrical stimulation of the barrel cortex, and this suppression at the collicular level was also evident in simultaneously recorded DA neurons. These correlated changes imply that interactions between stimulus types at the single-cell level in the colliculus are being passed on to DA neurons in the SNc by virtue of the fact that they share a common channel of communication.

### Functional Implications

Taken together, evidence that single-pulse electrical stimulation of the (barrel) cortex produces responses in previously unresponsive DA neurons when the SC is disinhibited with bicuculline, and muscimol-induced inhibition of the SC blocks or attenuates responses in DA neurons elicited by pulse trains applied to the barrel cortex, strongly suggests that the cortex can transmit information to DA neurons via the SC. As a consequence, this route of information flow is a clear contender for the substrate that underlies the second phase of the response of DA neurons to complex visual stimuli reported by several authors in the monkey, which has a peak latency of ∼160 ms (Hudgins et al. 2009; Morris et al. 2004; Nomoto et al. 2010). Somatosensory information reaches the somatosensory cortex more quickly than visual information reaches the visual cortex (somatosensory: ∼10 ms; Ahissar and Arieli 2001; Di et al. 1990; Martin et al. 2006; Zhu et al. 2009; visual: 30–50 ms; Maunsell and Gibson 1992; Thorpe and Fabre-Thorpe 2001), and so cortically driven responses to somatosensory stimuli have the capacity to be even faster than those to visual stimuli. Given dopamine's critical role in reinforcement learning (Wise 2004), we have argued that the short latency of the dopamine signal is particularly well suited to reinforce the discovery of agency—those events in the world for which the agent is responsible (Redgrave et al. 2008; Redgrave and Gurney 2006). According to this view, the advantage of having DA reinforcement occur before any behavior evoked by the sensory event itself is that the record of behavioral output (motor efference copy) will remain uncontaminated by noncausal components of behavior. This would greatly simplify the problem of assigning credit to appropriate causal components of immediately preceding behavior (Izhikevich 2007). Sensory cortical inputs to the DA systems—which have the potential to be modified by reward (see, e.g., Frankó et al. 2010; Hickey et al. 2010; Hui et al. 2009; Mogami and Tanaka 2006; Serences and Saproo 2010; Weil et al. 2010)—can be seen as a means by which the connection between an individual's behavior and its outcome is “stamped in.”

If the SC is the conduit used by the sensory cortex to evoke short-latency phasic responses in DA neurons, the question arises as to why this differs from the manner in which the cerebral cortex makes contact with other basal ganglia input nuclei. Thus with the evolutionary expansion of the cerebral cortex (Butler et al. 2011), direct, topographically organized connections were established with both the striatum (Nambu 2011; Selemon and Goldman-Rakic 1985) and the subthalamic nucleus (Nambu 2011; Nambu et al. 1997). Why not also with DA neurons in the ventral midbrain, which receive comparatively few direct cortical inputs (Frankle et al. 2006; Watabe-Uchida et al. 2012)? One possibility could be that the topographically organized cortico-striatal and cortico-subthalamic connections are designed to convey bids for behavioral selection (Redgrave et al. 1999), the competitions between which are resolved (Gurney et al. 2001; Humphries et al. 2006; Prescott et al. 2006) by selective disinhibition (Chevalier and Deniau 1990) within the basal ganglia's reentrant looped connectivity (Alexander et al. 1986). This highly topographic selection architecture may differ markedly from one that is designed to relay reinforcement signals widely throughout a selection network. Interactions within the striatum between the widely broadcast dopamine signal and more localized activity engendered by previously occurring successful bids for expression may be critical for reinforcement learning (Redgrave et al. 2008; Redgrave and Gurney 2006). The advantage of having early cortical processing access the basal ganglia's reinforcement mechanisms indirectly via the midbrain SC may be that the SC constitutes a useful point of convergence for many sources of sensory and nonsensory information (Edwards et al. 1979; May 2006) that the cortex needs to take into account when reinforcing behavioral output. Alternatively, viewing the various afferent inputs to the colliculus more equally, the SC may be viewed as providing a final common nodal point for information destined for DA neurons. This may be the substrate that accounts for the wide range of nonnoxious sensory stimuli that are able to produce responses in DA neurons at short latency in rats and other species. In rats, in addition to visual stimuli, auditory, whisker, and nonwhisker somatosensory stimuli all phasically activate DA neurons (Freeman and Bunney 1987; Kiyatkin 1988; Miller et al. 1981) and are all represented in the SC (Hemelt and Keller 2007; McHaffie et al. 1989; Vachon-Presseau et al. 2009). Given that the tectonigral projection is a conserved feature across rats, cats, and monkeys (Comoli et al. 2003: May et al. 2009; McHaffie et al. 2006), a similar system-level organization may be a widespread feature of the architecture for the regulation of DA neurons.

## GRANTS

This research was supported by grants from the Biotechnology and Biological Sciences Research Council (BB/D019648/1) and Wellcome Trust (080943 and 091409) (P. Redgrave and P. G. Overton).

## DISCLOSURES

No conflicts of interest, financial or otherwise, are declared by the author(s).

## AUTHOR CONTRIBUTIONS

Author contributions: C.B., L.D., L.W.B., N.V., and M.L. performed experiments; C.B., L.W.B., and S.H. analyzed data; C.B. and P.G.O. drafted manuscript; P.R. and P.G.O. conception and design of research; P.R. and P.G.O. interpreted results of experiments.
